# Sleep-EEG-based parameters for discriminating fatigue and sleepiness

**DOI:** 10.3389/frsle.2022.975415

**Published:** 2022-10-26

**Authors:** Koichi Fujiwara, Yuki Goto, Yukiyoshi Sumi, Manabu Kano, Hiroshi Kadotani

**Affiliations:** ^1^Department of Material Process Engineering, Nagoya University, Nagoya, Japan; ^2^Department of Systems Science, Kyoto University, Kyoto, Japan; ^3^Department of Psychiatry, Shiga University of Medical Science, Otsu, Japan

**Keywords:** EEG analysis, questionnaire survey, frequency analysis, biomarker identification, fatigue, sleepiness

## Abstract

Sleep quality can be evaluated from the viewpoint of recovery from fatigue and sleepiness; however, it is difficult to investigate sleep quality while distinguishing between the two. The aim of this study is to find biomarkers that can discriminate between daytime fatigue and sleepiness and to assess sleep quality in consideration thereof. We collected answers to questionnaires regarding daytime fatigue and sleepiness, as well as EEG data measured during sleep, from 754 city government employees in a rural area of Japan. The respondents were categorized into four groups in accordance with the severity of fatigue and sleepiness as assessed by the questionnaires: fatigued and sleepy (FS), fatigued, non-sleepy (FO), non-fatigued and sleepy (SO), and non-fatigued and non-sleepy (neither fatigued nor sleepy; NE) groups. EEG data of medial frontal electrodes were obtained with a one-channel portable electroencephalograph, and various sleep parameters such as powers or sleep durations in each stage were compared among the four groups. Statistical tests confirmed significant differences in some derived sleep parameters among the four groups. The Theta Delta power may be a biomarker that can discriminate between fatigue and sleepiness. In addition, the Delta and Theta powers may be associated with sleep quality in terms of recovery from sleepiness and fatigue, respectively. Moreover, high frequency or long duration of mid-arousals may contribute to recovery from fatigue. The results showed that fatigue and sleepiness have different effects on sleep, and multiple sleep parameters derived from EEG are associated with sleep quality.

## Introduction

Good sleep is essential for physical and cognitive performances, everyday health, and wellbeing (Spiegel et al., 1999; Maquet, 2001; Czeisler, 2015). Sleep quality should be taken into account in addition to sleep duration for evaluating good sleep (Pilcher et al., 1997). Complaints about sleep quality are common; epidemiological surveys indicate that 15–35% of the adult population complain of frequent sleep quality disturbance (Karacan et al., 1976; Bixler et al., 1979), and deterioration of sleep quality may be an important symptom of many sleep and medical disorders (Kripke et al., 1979).

Researchers have developed various methods for assessing sleep quality (Landry et al., 2015), which can be classified mainly into two types: subjective sleep quality assessment using questionnaires such as the Pittsburgh Sleep Quality Index (PSQI) (Buysse et al., 1989) and objective sleep quality measurement based on physiological measurements by means of polysomnography (PSG) (Åkerstedt et al., 1994; Littner et al., 2003). For example, total sleep duration, number of arousals, sleep efficiency, and duration of slow-wave sleep (SWS) have been used as objective indicators of sleep quality that can be derived from PSG (Unruh et al., 2008).

The objective measures are rarely consistent with the subjective assessments. Buysse et al. (Buysse et al., 2008) reported that the PSQI does not correlate well with PSG. It is difficult to rigorously evaluate “sleep quality" despite it being widely used in sleep medicine as well as in daily life.

In this study, we focus on daytime fatigue and sleepiness to evaluate sleep quality because recovery therefrom is one of the main functions of sleep. The close relationship between fatigue and sleep is generally known (Aaronson et al., 1999). For example, subjects with daytime fatigue show significantly low sleep efficiency (Stores et al., 1998; Jackson and Bruck, 2012).

Fatigue has been confused frequently with sleepiness since the symptom of fatigue is a poorly defined subjective feeling. Some studies suggest differences in sleep characteristics between daytime fatigue and sleepiness. Lichstein et al. reported that fatigue was associated with being female, a smoker, a high body-mass index (BMI), and low sleep efficiency, whereas sleepiness was not (Lichstein et al., 1997). Neu et al. showed that subjects with fatigue and without sleepiness had different electroencephalogram (EEG) powers in non-REM sleep from subjects with only sleepiness (Neu et al., 2014). In order to precisely assess sleep quality while avoiding confusion between fatigue and sleepiness, new biomarkers that can discriminate between daytime fatigue and sleepiness are required.

In this study, we analyze answers to questionnaires regarding fatigue and sleepiness, as well as sleep EEG data, collected from adult employees to find biomarkers that discriminate between fatigue and sleepiness. We employed a 1-channel portable electroencephalograph for EEG measurement because it is suitable for collecting EEG data from a large number of people. In addition, sleep parameters that affect sleep quality are discussed from the viewpoint of recovery of daytime fatigue and sleepiness.

## Methods

### Participants

**Figure 1:**
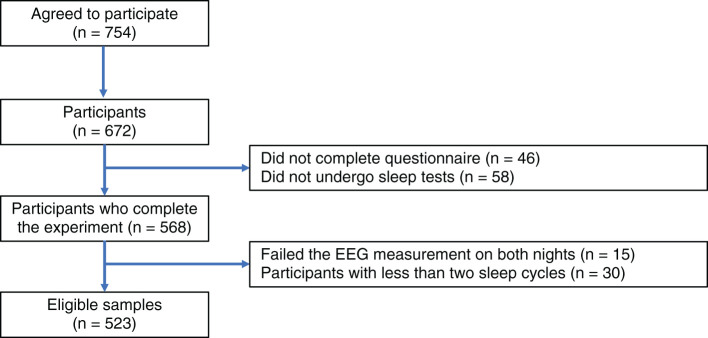
Study inclusion flowchart. The exclusion criteria in this study were (a) Participants who did not participate in the questionnaires and the sleep tests; (b) Participants who failed the EEG measurement on both nights; and (c) Participants with less than two sleep cycles.

We performed a questionnaire survey and sleep EEG measurement over two nights in 2018 and 2019 as part of the Night in Japan Home Sleep Monitoring (NinJaSleep) study (Takami et al., 2018; Omichi et al., 2022). All the participants in this study were government employees of Koka City, a rural city in Shiga prefecture, Japan. 754 employees agreed to participate in the study and the number of actual participants was 672 (288 males and 384 females, aged 20–72 years). The exclusion criteria were as follows: (a) Participants who did not participate in the questionnaires and the sleep tests; (b) Participants who failed the EEG measurement on both nights; and (c) Participants with less than two sleep cycles. According to these criteria, 523 subjects were retained for further analysis. Figure 1 shows a study inclusion flowchart.

The Ethics Committee of Shiga University of Medical Science approved the study protocol (R2017-111). The study was registered at UMIN-CTR (UMIN000028675, registered on August 15, 2017) and ClinicalTrials.gov (NCT03276585, registered on August 3, 2017). Informed consent was obtained from each participant prior to participation. The datasets analyzed in the current study are available from the corresponding author upon reasonable request.

### Measurements

All participants completed the Japanese version of the Chalder Fatigue Scale (CFS) (Chalder et al., 1993; Tanaka et al., 2008) and the Epworth Sleepiness Scale (ESS) (Johns, 1991; Takegami et al., 2009), and other general questionnaires about clinical characteristics before the sleep test. The CFS is a self-administered questionnaire for measuring the degree and severity of fatigue, consisting of 14 questions related to physical and mental fatigue. Each question is answered on a four-point scale ranging from asymptomatic to maximum symptomology: “0: Less than usual,” “1: as usual,” “2: More than usual,” and “3: Much more than usual.” Thus, the total score of the CFS ranges from 0 to 42. Since the CFS assesses fatigue over the past month, it is not affected by daily external factors. The Japanese version of CFS was validated by Tanaka et al. (2008). Supplementary Table S1 shows the original and the Japanese version of CFS.

The ESS is a self-administered questionnaire for measuring the degree of daytime sleepiness, which has been widely used for sleepiness assessment in both clinical and epidemiological settings. The ESS consists of eight questions, and respondents are asked to rate each on a four-point scale (0–3). The total ESS score ranges from 0 to 24. Since the ESS assesses daytime sleepiness in recent daily life, it is not affected by daily external factors. Takegami et al. (2009) made and validated the Japanese version of ESS.

The participants obtained all-night sleep EEG measurement from bedtime to wake-up time for two nights at home using a portable EEG device equipped with a single EEG channel with a sampling rate of 128 Hz (SLEEP SCOPE; Sleep Well Co., Japan). SLEEP SCOPE has been used in several sleep studies (Svensson et al., 2019; Liang and Chapa-Martell, 2021; Torimoto et al., 2022) and validated with medical EEG devices (Yoshida et al., 2015; Matsuo et al., 2016; Kawamura et al., 2022). An EEG electrode was positioned at the median frontal lobe, and the reference was the right mastoid bone. The participants attached and detached the electrodes by themselves. Overnight hospitalization was not needed, and the sleep tests were performed at the participants' homes using the portable EEG device.

### Subject groups classification based on questionnaires

We defined a CFS score of 16 or greater as fatigued and an ESS score of 8 or greater as sleepy. These cut-off values were determined using a median-split method (Waters et al., 2013; Matsuo et al., 2016). All subjects were classified into four groups as follows: the fatigued and sleepy (FS) group, the fatigued and non-sleepy (only fatigued; FO) group, the non-fatigued and sleepy (only sleepy; SO) group, and the non-fatigued and non-sleepy (neither fatigued nor sleepy; NE) group.

### EEG data analysis

In this study, EEG data obtained on the second night were analyzed to assess the first night effect, which is change that may occur in sleep structures caused by the sleep test itself. The EEG data of the first night were used when technicians determined that the EEG data of the second night were incorrectly measured.

The parameters representing sleep characteristics were extracted from the collected EEG data using two methods: time-domain analysis conducted by the technicians and frequency-domain analysis conducted by a computer.

**Figure 2:**
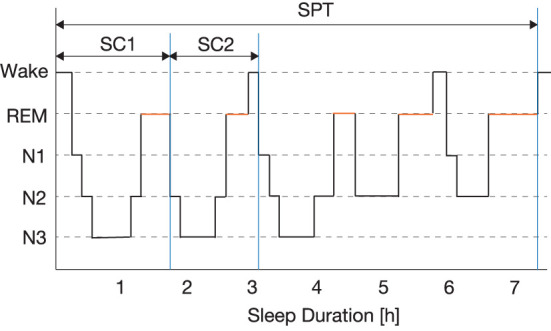
Definitions of the first sleep cycle (SC1), the second sleep cycle (SC2), and the whole sleep period time (SPT).

In the time-domain analysis, technicians scored the EEG data following the American Academy of Sleep Medicine (AASM) manual (AASM, 2014), and we derived 28 sleep parameters listed in Supplementary Table S2 based on the scoring result. In these parameters, the first sleep cycle (SC1) and the second sleep cycle (SC2) are defined as the period from sleep onset to the end of the first REM sleep, and the period from the end of the first REM sleep to the end of the second REM sleep, respectively. These definitions are illustrated in Figure 2.

The previous studies reported that slow wave sleep (SWS) is associated with fatigue (Buguet et al., 1980; Susić and Kovacévić-Ristanović, 1980) and SWS abundantly appears in the first half of the whole sleep (Kryger et al., 2022). In addition, early sleep stages are more important than later ones from the viewpoint of recovery of fatigue (Van Cauter and Plat, 1996). Although the last half of sleep such as SC3 and SC4 may be associated with sleep disorders; however, we excluded patients with sleep disorders in this study. In addition, it is difficult to analyze SC3 and SC4 due to their short duration. Thus, we focused on SC1 and SC2 in this study.

The frequency-domain analysis extracts sleep parameters based on the powers of EEG. To calculate accurate parameters, the following preprocessing is performed: (1) filter artifacts from the collected EEG data, (2) estimate power spectral density (PSD) from the filtered EEG data, (3) calculate the powers in four frequency bands based on the estimated PSD, and (4) filter spikes from the calculated powers. In step 3, the PSD is divided into four frequency bands: the Delta power (1–4 Hz), the Theta power (4–8 Hz), the Alpha power (8–13 Hz), and the Beta power (13–30 Hz). This preprocessing is described in detail in the Appendix.

After preprocessing, the filtered powers are clipped from the following three periods: SC1, SC2, and the whole sleep period time (SPT). The following eight power indexes are calculated in each period:

- Absolute power (abs): the absolute power in the four frequency bands (absDelta, absTheta, absAlpha, and absBeta).
- Relative power (rel): the power ratio of each frequency band and the total bandwidth (relDelta, relTheta, relAlpha, and relBeta).

Finally, nine types of sleep parameters described in Supplementary Table S3, such as maxVal (maximum value), maxT (timing with the maximum value), and Avg (average), are calculated based on each power index; that is, a total of 216 (= 3 periods × 8 power indexes × 9 types) parameters were extracted.

### Statistical analysis

The sleep parameters derived from both time-domain and frequency-domain analyses were compared among the four groups—FS, FO, SO, and NE groups. Since the Shapiro-Wilk test showed that the derived sleep parameters violated normality, we used the Kruskal-Wallis (KW) test, which is a non-parametric version of the one-way analysis of variance (ANOVA) that tests whether sample groups originate from the same distribution. The KW test is used for comparing between two or more independent groups consisting of equal or different sample sizes. Its null hypothesis is that the medians of all groups are equal, and the alternative hypothesis is that a median calculated from one group is different from that of at least one other group. In addition, we used the Dunn's test as a sub-effect test, which is used for multiple comparisons using rank sums. The significance level was set to *p* < 0.05.

The EEG data were analyzed using Python 3.6.4 with NumPy 1.16.2 and SciPy 1.1.0, and the statistical tests were performed in Python 3.6.4 with SciPy 1.1.0. Figures were plotted with Matplotlib 2.1.2.

## Results

### Subject groups

**Figure 3:**
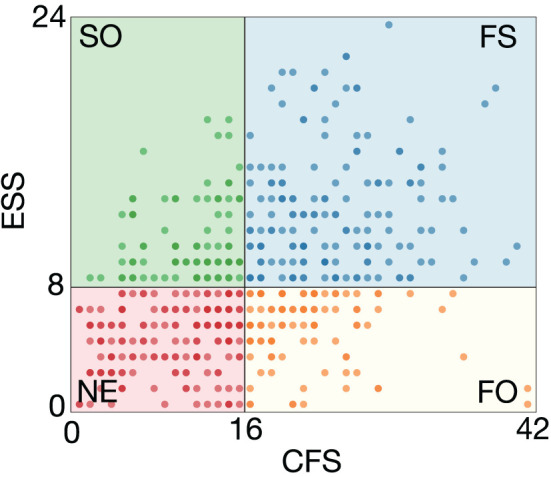
Scatter plot showing distribution of CFS and ESS scores. One hundred sixty-four subjects were categorized into the FS group, 98 into the FO group, 86 into the SO group, and 175 into the NE group. Colors denote the subject group.

One hundred and sixty four subjects were categorized into the FS group, 98 into the FO group, 86 into the SO group, and 175 into the NE group. Figure 3 shows a scatter plot of the distribution of the CFS and the ESS scores, wherein the colors denote the subject group. There was a weak positive correlation between the CFS and the ESS scores (*r* = 0.393, *p* < 0.001).

The biographic data of each group are displayed in Table 1. There was a significant difference in gender among the groups according to the χ^2^ test (*p* < 0.05), with the most significant proportion of females in the FS group and a relatively large proportion of males in the NE group. The KW test showed a significant difference in age among the groups (*p* < 0.05). Multiple comparisons by the Dunn's test showed that the NE group was significantly older than the SO and FS groups, and the FO group was also significantly older than the SO and FS groups (*p* < 0.05). These results showed that groups with sleepiness were significantly younger than groups without sleepiness, and sleepiness increased with a decrease in age (*r* = −0.236, *p* < 0.001). A significant difference in BMI was not confirmed.

**Table 1:** Demographic data of each group.

	**FS**	**FO**	**SO**	**NE**	***p***
Num of subjects	164	98	86	175	
Male/Female	49/115	33/65	30/56	86/89	0.002 (χ^2^ test)
Age (mean ± SD)	42.2 ± 10.9	46.9 ± 9.5	41.5 ± 12.1	48.2 ± 12.4	< 0.001 (KW test)
BMI (mean ± SD)	22.3 ± 3.7	23.3 ± 5.2	22.2 ± 3.2	22.6 ± 3.8	0.195 (KW test)

### Sleep parameter comparison

The KW test was conducted on 244 sleep parameters derived from the analysis of the time-domain (28 parameters) and the frequency-domain (216 parameters). Thirty-one out of the 244 sleep parameters listed in Table 2 had significant differences (*p* < 0.05). Of the 31 parameters, eight were derived from the time domain analysis and 23 were derived from the frequency domain analysis—specifically, 14 from SC1, three from SC2, and six from SPT. Multiple comparisons were subsequently conducted using the Dunn's test. The *p*-values calculated for all group pairs are shown in Table 3. The pairs with significant differences (*p* < 0.05) are colored gray. Most of these parameters had significant differences between the FS and NE groups, while only WASO2h (wake time after sleep onset 2 h before the final awakening) was significantly different between the FO and the SO groups.

**Table 2:** Significant parameters confirmed by KW test (*p* < 0.05).

**Method**	**Period**	**Power index**	**Parameter**	**Groups (mean** **±SD)**				**KW test**
				**FS**	**FO**	**SO**	**NE**	***p*-value**
Time-domain			TSI	52.9 ± 22.1	51.6 ± 27.0	55.3 ± 22.1	59.4 ± 25.0	0.011
			TS3	31.3 ± 29.4	27.4 ± 26.3	34.8 ± 29.9	25.5 ± 28.6	0.036
			%SI	14.8 ± 5.9	14.3 ± 6.8	15.0 ± 5.2	15.6 ± 5.6	0.047
			%S3	8.46 ± 7.63	7.59 ± 7.08	9.37 ± 8.05	6.91 ± 8.11	0.02
			%SW	6.96 ± 3.92	6.67 ± 4.17	7.13 ± 4.49	8.25 ± 5.31	0.017
			ARI	8.36 ± 4.70	8.00 ± 5.01	8.56 ± 5.39	9.90 ± 6.37	0.017
			WASO	25.4 ± 15.7	25.1 ± 19.8	26.8 ± 20.1	32.5 ± 23.5	0.002
			WASO2h	13.5 ± 9.5	13.0 ± 10.8	14.8 ± 11.3	16.2 ± 12.7	0.005
Frequency-domain	SCI	absDelta	maxVal	213.0 ± 128.0	200.3 ± 95.2	245.4 ± 169.4	197.5 ± 152.8	0.023
			minVal	49.0 ± 35.7	45.8 ± 32.1	52.3 ± 48.1	40.3 ± 32.5	0.031
			minT	171.1 ± 75.7	179.4 ± 86.4	188.4 ± 78.4	192.3 ± 82.1	0.016
			Avg	137.1 ± 80.9	129.3 ± 59.6	153.2 ± 102.1	126.8 ± 97.3	0.039
			Std	51.1 ± 35.3	47.7 ± 27.7	60.6 ± 43.6	47.1 ± 39.6	0.019
		relDelta	minVal	0.61 ± 0.11	0.61 ± 0.09	0.60 ± 0.12	0.58 ± 0.11	0.023
			minT	166.6 ± 74.6	171.9 ± 84.0	184.5 ± 77.4	182.4 ± 78.3	0.027
		relTheta	maxVal	0.21 ± 0.05	0.21 ± 0.04	0.21 ± 0.04	0.22 ± 0.04	0.003
			Avg	0.15 ± 0.03	0.15 ± 0.03	0.15 ± 0.03	0.16 ± 0.03	0.027
			Total	28.1 ± 14.0	29.7 ± 16.0	30.8 ± 13.8	32.3 ± 14.6	0.012
		absBeta	maxT	46.6 ± 66.5	69.7 ± 98.1	73.3 ± 86.6	58.0 ± 75.8	0.023
			maxTratio	0.28 ± 0.38	0.36 ± 0.42	0.38 ± 0.42	0.30 ± 0.40	0.043
		relBeta	maxT	153.5 ± 89.7	168.0 ± 101.8	169.3 ± 105.8	180.4 ± 92.2	0.046
			Total	7.16 ± 4.55	8.07 ± 7.03	7.91 ± 5.96	8.30 ± 4.92	0.047
	SC2	absDelta	minVal	25.2 ± 16.1	23.5 ± 11.1	25.0 ± 13.6	21.5 ± 12.2	0.017
		relDelta	minVal	0.60 ± 0.11	0.59 ± 0.09	0.58 ± 0.12	0.57 ± 0.10	0.047
		relTheta	minTratio	0.83 ± 0.22	0.85 ± 0.23	0.89 ± 0.19	0.88 ± 0.20	0.04
	SPT	absDelta	maxVal	227.6 ± 125.3	210.4 ± 95.6	255.4 ± 168	211.9 ± 153.8	0.037
			Std	54.2 ± 33.3	49.6 ± 24.6	61.2 ± 42.1	51.2 ± 42.8	0.042
		relTheta	minTratio	0.82 ± 0.22	0.83 ± 0.18	0.79 ± 0.24	0.75 ± 0.24	0.047
			Total	123.1 ± 31.8	124.1 ± 31.3	127.7 ± 34.2	134.6 ± 35.5	0.011
		relAlpha	minT	593.6 ± 201.3	609.4 ± 221.2	625.9 ± 186.9	656.3 ± 187.4	0.029
		relBeta	minT	520.8 ± 227.0	552.7 ± 245.1	539.5 ± 206.1	597.5 ± 212.3	0.011

**Table 3:** Multiple comparison results between groups by the Dunn's test.

**Method**	**Period**	**Power index**	**Parameter**	***p*** **-value**					
				**FS-FO**	**FS-SO**	**FS-NE**	**FO-SO**	**FO-NE**	**SO-NE**
Time-domain			TSI	0.312	0.380	0.015	0.096	0.002	0.265
			TS3	0.531	0.423	0.027	0.206	0.202	0.008
			%SI	0.194	0.521	0.098	0.089	0.006	0.471
			%S3	0.608	0.497	0.013	0.291	0.104	0.006
			%SW	0.314	0.880	0.024	0.315	0.003	0.087
			ARI	0.314	0.866	0.023	0.306	0.003	0.089
			WASO	0.273	0.764	0.004	0.224	< 0.001	0.039
			WASO2h	0.243	0.140	0.015	0.020	0.001	0.600
Frequency-domain	SCI	absDelta	maxVal	0.994	0.223	0.042	0.275	0.078	0.004
			minVal	0.989	0.771	0.014	0.784	0.036	0.020
			minT	0.620	0.048	0.004	0.177	0.044	0.676
			Avg	0.964	0.368	0.040	0.440	0.070	0.009
			Std	0.957	0.094	0.085	0.120	0.153	0.002
		relDelta	minVal	0.611	0.590	0.004	0.963	0.045	0.062
			minT	0.874	0.026	0.016	0.062	0.056	0.787
		relTheta	maxVal	0.408	0.150	< 0.001	0.559	0.022	0.123
			Avg	0.302	0.295	0.003	0.958	0.119	0.151
			Total	0.595	0.108	0.002	0.321	0.031	0.336
		absBeta	maxT	0.024	0.006	0.239	0.601	0.204	0.072
			maxTratio	0.032	0.017	0.440	0.762	0.133	0.075
		relBeta	maxT	0.458	0.110	0.007	0.423	0.111	0.530
			Total	0.577	0.477	0.007	0.874	0.076	0.127
	SC2	absDelta	minVal	0.621	0.975	0.004	0.69	0.048	0.019
		relDelta	minVal	0.133	0.173	0.005	0.942	0.368	0.345
		relTheta	minTratio	0.162	0.022	0.010	0.393	0.421	0.852
	SPT	absDelta	maxVal	0.633	0.402	0.029	0.243	0.163	0.008
			Std	0.751	0.277	0.051	0.210	0.173	0.007
		relTheta	minTratio	0.674	0.273	0.007	0.532	0.058	0.263
			Total	0.697	0.364	0.002	0.63	0.019	0.089
		relAlpha	minT	0.475	0.295	0.003	0.743	0.070	0.171
		relBeta	minT	0.257	0.641	0.001	0.576	0.103	0.029

Colored cells denote significant differences (*p* < 0.05).

### Time domain parameters

**Figure 4:**
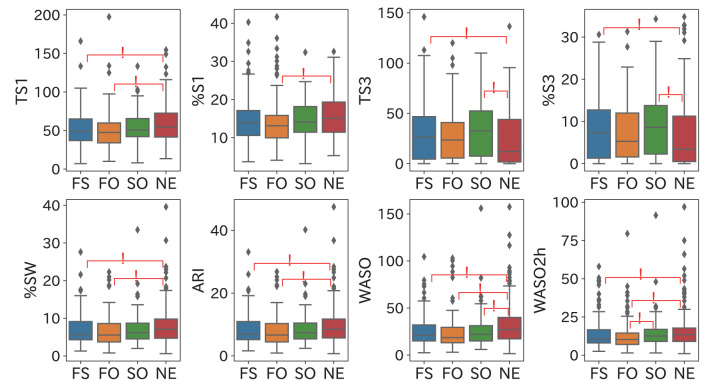
Comparison results of significant time-domain sleep parameters. These results suggest the following points: fatigue reduces N1 duration, sleep extends N3 duration, and the mid-arousal duration in two hours before the final awakening is decreased by fatigue and increased by sleepiness. Significant group pairs (*p* < 0.05) are marked with an asterisk.

Figure 4 shows the comparison results of the significant time-domain sleep parameters. The following three major results were obtained.

- N1 and fatigue
  TS1 (time in N1) was significantly smaller in the FS and FO groups than in the NE group, and %S1 (percentage of N1) was significantly smaller in the FO group than in the NE group. These results suggest that fatigue reduces N1 duration.
- N3 and sleepiness
  TS3 (time in N3) and %S3 (percentage of N3) were significantly larger in the FS and SO groups than in the NE group, suggesting that sleepiness is associated with the extension of N3 duration.
- Mid arousals and fatigue/sleepiness
  %SW (percentage of wake) and ARI (arousal index) were significantly lower in the FS and FO groups than in the NE group, which suggests that fatigue decreased the percentage and the frequency of mid arousal time. WASO (wake time after sleep onset) was significantly smaller in the FS, FO, and SO groups than in the NE group. A significant difference between the SO and NE groups was not confirmed in %SW; however, there was a significant difference in WASO. The SO group had a relatively shorter SPT than the NE group and a small WASO. WASO2h was significantly smaller in the FS and FO groups than in the NE group, and in the FO group than in the SO group, which suggests that the total mid-arousal duration 2 h before the final awakening is decreased by fatigue and increased by sleepiness.

### Frequency domain parameters

**Figure 5:**
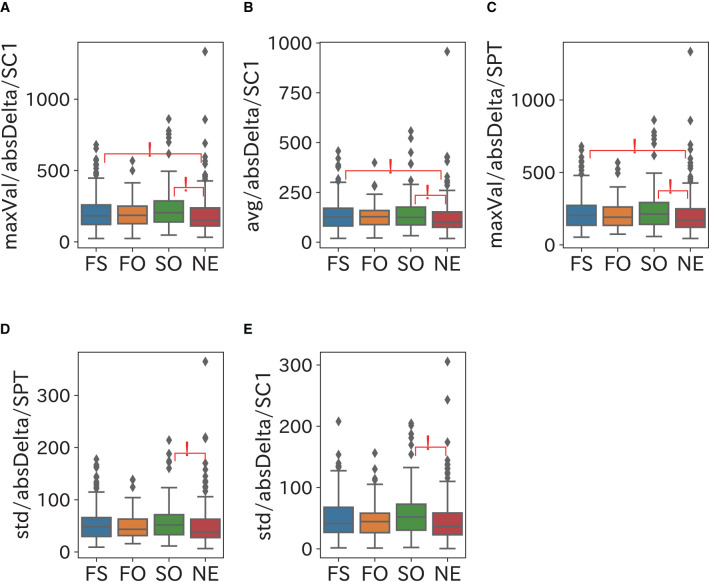
Relationship between Delta power and sleepiness. Sleepiness may increase the maximum and the mean of the Delta power in SC1 and its variability in both SC1 and SPT. **(A–C)** maxVal (maximum value) in SC1 and Avg (average value) and maxVal in SPT of absDelta were significantly larger in the FS and SO groups than in the NE group. The maxVal range in SC1 and SPT were almost the same and SPT contained SC1, which suggests that most subjects achieved the maximum of the Delta power during SC1. **(D,E)** Std (standard deviation) of absDelta in SC1 and SPT were significantly larger in the SO group than in the NE group.

**Figure 6:**
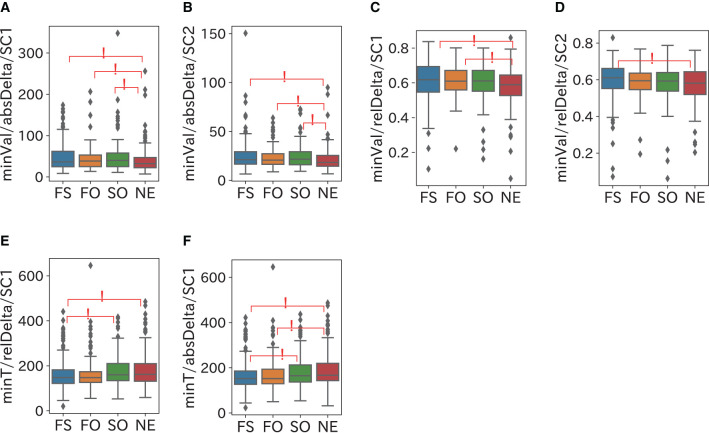
Relationship between Delta power and fatigue. The Delta power in SC1 becomes large and reaches the minimum value at early timing when a participant is fatigued. **(A,B)** minVal (minimum value) of absDelta in SC1 and SC2 were significantly larger in the FS and FO groups than in the NE group. **(C)** relDelta (relative Delta power) in SC1 was significantly larger in the FS and FO groups than in the NE group. **(D)** relDelta in SC2 was significantly larger in the FS group than in the NE group. **(E)** minT (timing with the minimum value) of relDelta in SC1 was significantly longer in the SO and NE groups than in the FS group. **(F)** minT of absDelta in SC1was significantly longer in the SO and NE groups than the FS group and in the NE group than the FO group.

**Figure 7:**
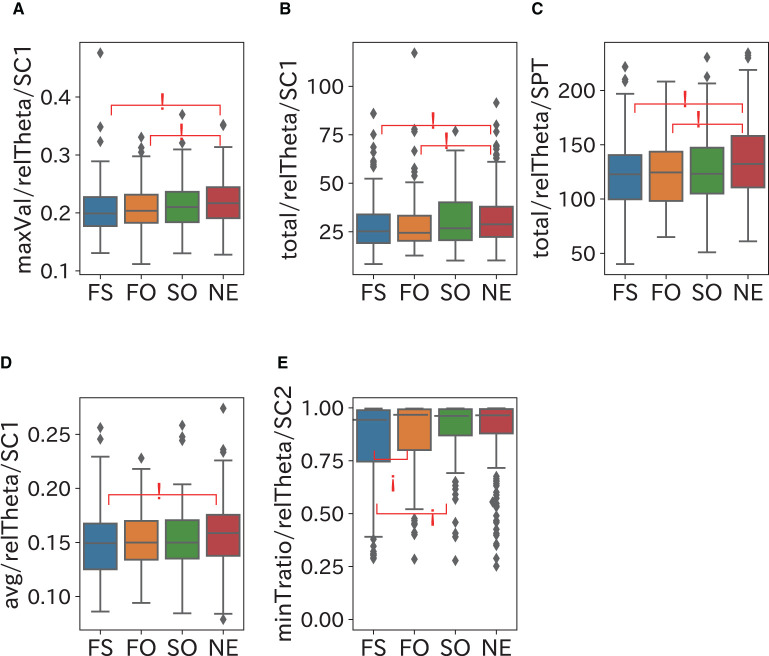
Relationship between Theta power and fatigue. The Theta powers in SC1 and SPT become small and the Theta power in SC2 reaches minimum early when a participant is fatigued. **(A–C)** maxVal in SC1 and total in SPT of relTheta (relative Theta power) were significantly smaller in the FS and FO groups than in the NE group. **(D)** Avg in SC1 of the FS group was significantly smaller than of the NE group. **(E)** minTratio (ratio of minT to the length of the period) of relTheta in SC2 was significantly larger in the SO and NE groups than in the FS group.

**Figure 8:**
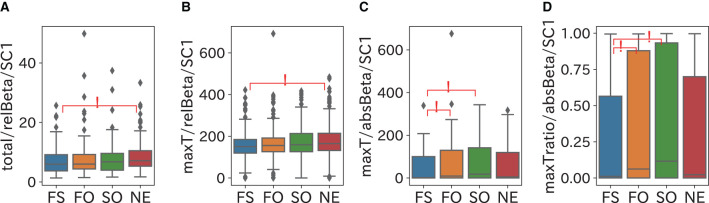
Relationship between Beta power and fatigue and sleepiness. At least fatigue or sleepiness contributes to a decrease in the Beta power in SC1, and the combination of fatigue and sleepiness leads to the Beta power in SC1 reaching its maximum early. **(A)** Total (sum of power) of relBeta (relative Beta power) in SC1 was significantly smaller in the FS group than in the NE group. **(B)** maxT of relBeta in SC1 was significantly smaller in the FS group than in the NE group. **(C,D)** maxT (timing with the maximum value) and maxTratio (ratio of maxT to the length of the period) of absBeta (absolute Beta power) in SC1 were significantly larger in the FO and SO groups than in the FS group.

The comparison results of the frequency domain sleep parameters in which a significant difference was confirmed are shown in Figures 5–8. We obtained the following relationships of fatigue and sleepiness with parameters of the Delta, the Theta, and the Beta powers.

- Delta power parameters
  As to absDelta (absolute Delta power), maxVal (maximum value) in SC1 and Avg (average value) and maxVal in SPT were significantly larger in the FS and SO groups than in the NE group, as shown in Figures 5A–C. The maxVal range in SC1 and SPT were almost the same and SPT contained SC1, which suggests that most subjects got the maximum of the Delta power during SC1. In addition, Std (standard deviation) of absDelta in SC1 and SPT were significantly larger in the SO group than in the NE group according to Figures 5D,E. Thus, sleepiness may increase the maximum and the mean of the Delta power in SC1 and its variability in both SC1 and SPT.
  minVal (minimum value) of absDelta in SC1 and SC2 were significantly larger in the FS and FO groups than in the NE group, as shown in Figures 6A,B. relDelta (relative Delta power) in SC1 was significantly larger in the FS and FO groups than in the NE group (Figure 6C), and relDelta in SC2 was significantly larger in the FS group than in the NE group (Figure 6D). According to Figures 6E,F, minT (timing with the minimum value) of relDelta in SC1 was significantly longer in the SO and NE groups than in the FS group, and minT of absDelta in SC1was also significantly longer in the SO and NE groups than in the FS group, and in the NE group than in the FO group. These results suggest that the Delta power in SC1 becomes large, and subsequently reaches the minimum value at an early timing when the participant is fatigued.
- Theta power parameters
  Figure 7 shows the relationship between fatigue and the Theta power. Regarding relTheta (relative Theta power), maxVal in SC1 and Total in SPT were significantly smaller in the FS and FO groups than in the NE group (Figures 7A–C), and the Avg in SC1 of the FS group was significantly smaller than that of the NE group (Figure 7D). Thus, the maximum, the mean, and the Total of the Theta power in SC1 and SPT may decrease with fatigue. As shown in Figure 7E, minTratio (ratio of minT to the length of the period) of relTheta in SC2 was significantly larger in the SO and NE groups than in the FS group. These results suggest that the Theta powers in SC1 and SPT become small and the Theta power in SC2 reaches minimum early when a participant is fatigued.
- Beta power parameters
  As to relBeta (relative Beta power), Total (sum of power) in SC1 was significantly smaller in the FS group than in the NE group, as shown in Figure 8A, which suggests that at least fatigue or sleepiness contributes to a decrease in the Beta power in SC1. maxT of relBeta in SC1 was significantly smaller in the FS group than in the NE group, as shown in Figure 7B. In addition, Figures 8C,D show that maxT (timing with the maximum value) and maxTratio (ratio of maxT and the length of the period) of absBeta (absolute Beta power) in SC1 were significantly larger in the FO and SO groups than in the FS group. These results indicate that the combination of fatigue and sleepiness leads to the Beta power in SC1 reaching its maximum early.

### Effect question about sleepiness

The third question of CFS asks about sleepiness; “Do you feel sleepy or drowsy?” We removed this question in order to completely separate sleepiness and fatigue when the participates were categorized into four groups.

The cut-off value of CFS was 14 when the third question was removed since it was defined based the median-split method (Waters et al., 2013). The numbers of participants in FS, FO, SO, and NE were 169, 113, 81, and 160, which shows that the third question of CFS did not significantly affect grouping (the χ^2^ test, *p* = 0.21).

## Discussion

In this study, we analyzed the sleep EEG data by means of time-domain analysis and frequency-domain analysis by categorizing participants into four groups in accordance with the CFS and the ESS: the fatigued and sleepy (FS) group, the fatigued and non-sleepy (only fatigue; FO) group, the non-fatigued and sleepy (only sleepy; SO) group, and the non-fatigued and non-sleepy (neither fatigue nor sleepy; NE) group. Our analysis results specified some sleep parameters that are different between fatigue and sleepiness.

N3 sleep, also known as slow-wave (Delta wave) sleep, is the deepest sleep stage dominated by Delta waves and appears mostly in SC1. The time-domain analysis indicated that sleepiness might contribute to extending the N3 duration. According to the frequency-domain analysis, sleepiness increases the maximum, the mean, and the variability of the Delta power in SC1, and extends the duration of N3 in SC1. Our data showed that the increase in the Delta power extends the N3 duration in SC1 when a participant is sleepy, which is consistent with a previous study which indicated that Delta waves are associated with the depth of sleep and sleepiness (Borbély et al., 1981). On the other hand, our data showed slightly weak negative correlations between age and the N3 duration (*r* = −0.164, *p* < 0.001) and the mean Delta power (*r* = −0.187 *p* < 0.001), which is consistent with a previous research which indicated that the duration of N3 decreases with age (Luca et al., 2015).

We introduce ΔPower as the difference between maxVal and minVal of the powers. The ΔDelta power in SC1 may increase when participants are sleepy because it was confirmed that the maximum and the variability of the Delta power in SC1 increase with sleepiness. Additional analyses were performed to verify our hypothesis. The KW test and the Dunn's test showed that the ΔDelta power in SC1 was significantly larger in the SO group than in the NE group (*p* = 0.021). Thus, it was also confirmed that the ΔDelta power in SC1 increases with sleepiness. On the other hand, the ΔDelta power in SC1 may decrease with fatigue, since the Delta power stays high in SC1, and reach its minimum at an early timing in SC1 when a participant is fatigued.

This analysis indicates that the ΔDelta power may be a biomarker that can discriminate between sleepiness and fatigue since the ΔDelta power becomes small with fatigue and large with sleepiness in SC1. Thus, the ΔDelta power could be a helpful tool for evaluating sleepiness recovery.

Our analysis results also showed that fatigue shortens the N1 duration and the total mid-arousal duration and reduces the Beta power in SC1, while sleepiness extends the mid-arousal duration 2 h before final awakening. Previous studies have shown that a small Beta power is associated with a high arousal level and shallow sleep, which indicates that Beta waves are related to wakefulness (Perlis et al., 2001). Beta power is high in people with insomnia (Spiegelhalder et al., 2012) and depression (Nofzinger et al., 2000) who have problems in sleep quality. However, our analysis suggested that a low Beta power may improve sleep quality by shortening the N1 duration and the mid-arousal duration. Thus, it is concluded that a low Beta power in SC1 caused by fatigue may shorten the N1 and the mid arousal duration, while a high Beta power caused by sleepiness extends the mid-arousal duration 2 h before the final awakening.

Sleepiness raises the maximum of the Beta power and delays its timing 2 h before the final awakening, which means that sleepiness may raise the Beta power toward the final awakening. We compared seven sleep parameters, maxVal, maxT, minVal, minT, Avg, Std, and Total, of the Beta power derived from 2 h before the final awakening among the four groups. Statistical tests showed that only maxVal and maxT were significantly different, and both were significantly larger in the FS and SO groups than in the NE group (*p* < 0.05).

Although it is often considered that the frequency and the duration of mid-arousals reflect poor sleep quality, our analysis showed that they are associated with fatigue recovery. In addition, sleepiness may extend the mid-arousal duration 2 h before the final awakening by raising the Beta power. Thus, mid-arousals during the 2 h before final awakening may be a useful biomarker for distinguishing between fatigue and sleepiness.

Our analysis result suggest that fatigue decreases the Theta power, reflecting a homeostatic dysfunction of sleep. Fatigue decreases the maximum and the mean of the Theta power both in SC1 and SPT and moves the timing of the maximum of the Theta power forward in SC2. Theta waves are involved in homeostatic sleep processes and enhance sleepiness recovery (Finelli et al., 2000). Sleepiness results from impairment of sleep homeostasis and can be recovered when sleep homeostasis is repaired (Neu et al., 2014). Although Theta power has rarely been mentioned in assessment of sleep quality, a large Theta power may be associated with sleep quality in terms of fatigue recovery. In addition, the Theta power represents an essential difference in sleep characteristics between fatigue and sleepiness from the viewpoint of sleep homeostasis.

In our data, there was a significant difference in gender among the groups. The gender distribution was mostly consistent with a study that indicated fatigue and insomnia are twice as common in female as in male (Sharpe and Wilks, 2002; Lind et al., 2015). The groups with sleepiness were significantly younger than groups without sleepiness, which indicated that younger participants tend to have sleepiness than older participants in our data. According to a survey by the Ministry of Health, Labour and Welfare, Japan, daytime sleepiness decrease with age in the Japanese population (Ministry of Health LaW, 2019). Thus, the distributions of gender and age in our data are consistent with the previous reports.

Our analysis will contribute to finding a new method for detecting chronic fatigue syndrome. In addition, it may help to investigate pathophysiology and new therapies of diseases with strong fatigue like post-COVID-19 fatigue.

The limitations of this study include the collected data, such as all of the participants being Japanese and recruited at a single region, and the number of female participants was about twice that of male participants; that is, we could consider neither racial nor regional difference in this study. Therefore, we need to collect data from participants in various regions to confirm our results.

## Conclusion

We analyzed the sleep EEG data collected from a large population focusing on fatigue and sleepiness. Various sleep parameters were extracted from the EEG data with the time-domain analysis and the frequency-domain analysis and compared among participants groups categorized in accordance with their sleepiness and fatigue.

Our analysis showed that fatigue and sleepiness have different effects on sleep, particularly in the Delta power and Theta power. Sleep quality can be evaluated by using multiple sleep parameters such as the frequency and the duration of mid-arousals as well as parameters from sleep EEG.

In a future work, we will establish a new EEG-based sleep quality assessment method that can respectively evaluate sleepiness and fatigue.

## Data availability statement

The raw data supporting the conclusions of this article will be made available by the authors, without undue reservation.

## Ethics statement

The studies involving human participants were reviewed and approved by the Ethics Committee of Shiga University of Medical Science. The patients/participants provided their written informed consent to participate in this study.

## Author contributions

KF and YG designed the study, analyzed the data and wrote the manuscript. YS collected and organized the data. MK supervised the study and the analysis and edited the manuscript. HK supervised the study and acquired the fund for this study. All authors contributed to the article and approved the submitted version.

## Funding

This work was supported in part by a research grant from the Investigator-Initiated Studies Program of Merck Sharp and Dohme LLC/MSD K.K.. HK received grants from Eisai Co., Ltd., and SECOM Science and Technology Foundation, reports consulting fees from Takeda Pharmaceutical Co., Ltd., and associated with a laboratory that was supported by donations from Fukuda Lifetech Co., Ltd., Fukuda Life Tech Keiji Co., Ltd., Tanaka Sleep Clinic, Akita Sleep Clinic, and Ai Care Co., Ltd. to Shiga University of Medical Science. The funders had no role in the design of the study; in the collection, analyses, or interpretation of data; in the writing of the manuscript, or in the decision to publish the results.

## Conflict of interest

KF and MK were employed by Quadlytics Inc. The remaining authors declare that the research was conducted in the absence of any commercial or financial relationships that could be construed as a potential conflict of interest.

## Publisher's note

All claims expressed in this article are solely those of the authors and do not necessarily represent those of their affiliated organizations, or those of the publisher, the editors and the reviewers. Any product that may be evaluated in this article, or claim that may be made by its manufacturer, is not guaranteed or endorsed by the publisher.

## Figures and Tables

**Figure A1:**
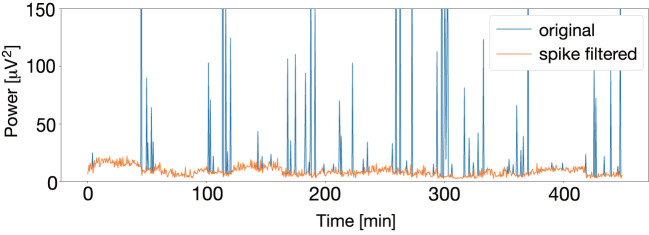
Calculated original power (blue) and power after spikes are filtered with the Hampel identifier (orange). The original power has many spikes because artifacts remain in the EEG data even when the filters are applied; however, spikes are successfully filtered with the Hampel identifier.

## Abbreviations

abs: absolute power
ANOVA: analysis of variance
ARI: arousal index
Avg: average
BMI: Body-Mass Index
CFS: the Chalder Fatigue Scale
EEG: Electroencephalogram
EES: Epworth Sleepiness Scale
FO group: Only Fatigued group
FS group: Fatigued and Sleepy group
KW test: Kruskal-Wall test
MAD: median absolute deviation
maxT: timing with the maximum value
maxTratio: ratio of maxT and the length of the period
maxVal: maximum value
minT: timing with the minimum value
minVal: minimum value
NE group: NEither fatigued nor sleepy group
PSD: Power Spectral Density
PSG: Polysomnography
PSQI: Pittsburgh Sleep Quality Index
REM: Rapid Eye Movement
rel: relative power
SC1: first Sleep Cycle
SC2: second Sleep Cycle
SO group: Only Sleepy group
SPT: Sleep Period Time
Std: Standard Deviation
SWS: Slow-Wave Sleep
TS1: time in N1
TS3: time in N3
WASO: wake time after sleep onset
WASO2h: wake time after sleep onset 2 h before the final awakening
%S1: percentage of N1
%S3: percentage of N3
%SW: percentage of wake.

## Appendix

This Appendix explains the preprocessing method of EEG data for the frequency-domain analysis. An appropriate preprocessing is needed because PSD calculation is intolerant of artifacts. The preprocessing procedure is as follows: (1) filter artifacts from the EEG data, (2) estimate PSD from the filtered EEG data, (3) calculate the powers in each frequency band based on the estimated PSD, and (4) filter spikes from the calculated powers.

1) Artifact filtering

Abandpass filter of 1–32 Hz is applied to attenuated relatively regular waves, such as lead impedance, heart, and respiratory artifacts. In addition, the 30-s epoch EEG data with an amplitude over ±100 μV are removed as artifacts because the maximum voltage of EEG is around ± 75 μV, even in N3 which has the largest amplitude in the entire sleep. Such high amplitude artifacts may originate from body movement or bruxism.

2) PSD estimation

We estimate PSD by using Welch's method (Neu et al., 2014) from the filtered EEG data using 15 overlapping 4-s sub-epochs for each 30-s epoch, with a 50% Hann window.

3) Power calculation

Based on the estimated PSD, the powers in four frequency bands commonly used in sleep science are calculated: the Delta power (1–4 Hz), the Theta power (4–8 Hz), the Alpha power (8–13 Hz), and the Beta power (13–30 Hz).

4) Spike filtering

Figure A1 is an example of the calculated power in step 3, which shows that it contains many spikes because artifacts remain in the EEG data even when the filters are applied in step 1. To eliminate these spikes, the Hampel identifier (Liu et al., 2004) is used, which is a variation of the three-sigma rule in statistics for improving robustness against outliers. When a sequence *x*_1_, *x*_2_, ⋯*x*_*n*_, ⋯  and a sliding window of length *k* are given, a point-to-point median *m*_*i*_ and a standard deviation σ_*i*_ can be estimated as follows:

(A1)
$$\begin{array}{l} m_{i}=\text{median}(x_{i-k},x_{i-k+1},x_{i-k+2},\;\cdots ,\;x_{i},\;\cdots ,\;x_{i+k-2},\\ \;x_{i+k-1},\;x_{i+k}) \end{array}$$

(A2)
$$\begin{array}{lll} \sigma_{i} & = & 1.4826\times \text{MAD}. \end{array}$$

MAD (median absolute deviation) is defined as

(A3)
$$\begin{array}{l} \text{MAD}=\text{median}\left(\left|x_{i-k}-m_{i}|,\;\cdots \;,\;|x_{i+k}-m_{i}\right|\right). \end{array}$$

When |*x*_*i*_−*m*_*i*_|>3σ_*i*_, the Hampel identifier detects *x*_*i*_ as an outlier and replaces it with *m*_*i*_. Figure A1 shows an application example of the Hampel identifier to the calculated power, which shows the spikes were successfully filtered. In this study, a sliding window of length *k* = 60 was adopted.

## References

[B1] Åkerstedt T. Hume K. Minors D. Waterhouse J. (1994). The meaning of good sleep: a longitudinal study of polysomnography and subjective sleep quality. J. Sleep Res. 3, 152–158. 10.1111/j.1365-2869.1994.tb00122.x 10607120

[B2] Aaronson L. S. Teel C. S. Cassmeyer V. Neuberger G. B. Pallikkathayil L. Pierce J.. (1999). Defining and measuring fatigue. Image J. Nurs. Sch. 31, 45–50. 10.1111/j.1547-5069.1999.tb00420.x 10081212

[B3] AASM (2014). The AASM Manual for the Scoring of Sleep and Associated Events: Rules, T.erminology, and Technical Specifications, Version 2.0.3.

[B4] Bixler E. O. Kales A. Soldatos C. R. Kales J. D. Healey S. (1979). Prevalence of sleep disorders in the Los Angeles metropolitan area. Am. J. Psychiatry. 136, 1257–1262. 10.1176/ajp.136.10.1257 314756

[B5] Borbély A. A. Baumann F. Brandeis D. Strauch I. Lehmann D. (1981). Sleep deprivation: effect on sleep stages and EEG power density in man. Electroencephal. Clin. Neurophysiol. 51, 483–495. 10.1016/0013-4694(81)90225-X 6165548

[B6] Buguet A. Roussel B. Angus R. Sabiston B. Radomski M. (1980). Human sleep and adrenal individual reactions to exercise. Electroencephal. Clin. Neurophysiol. 49, 515–523. 10.1016/0013-4694(80)90394-6 6158433

[B7] Buysse D. J. Hall M. L. Strollo P. J. Kamarck T. W. Owens J. Lee L.. (2008). Relationships between the Pittsburgh Sleep Quality Index (PSQI), Epworth Sleepiness Scale (ESS), and clinical/polysomnographic measures in a community sample. J. Clin. Sleep Med. 4, 563–571. 10.5664/jcsm.27351 19110886 PMC2603534

[B8] Buysse D. J. Reynolds C. F. Monk T. H. Berman S. R. Kupfer D. J. (1989). The Pittsburgh Sleep Quality Index: a new instrument for psychiatric practice and research. Psychiatry Res. 28, 193–213. 10.1016/0165-1781(89)90047-4 2748771

[B9] Chalder T. Berelowitz G. Pawlikowska T. Watts L. Wessely S. Wright D.. (1993). Development of a fatigue scale. J. Psychosom. Res. 37, 147–153. 10.1016/0022-3999(93)90081-P 8463991

[B10] Czeisler C. A. (2015). Duration, timing and quality of sleep are each vital for health, performance and safety. Sleep Health. 1, 5–8. 10.1016/j.sleh.2014.12.008 29073414

[B11] Finelli L. A. Baumann H. Borbély A. A. Achermann P. (2000). Dual electroencephalogram markers of human sleep homeostasis: correlation between theta activity in waking and slow-wave activity in sleep. Neuroscience. 101, 523–529. 10.1016/S0306-4522(00)00409-7 11113301

[B12] Jackson M. L. Bruck D. (2012). Sleep abnormalities in chronic fatigue syndrome/myalgic encephalomyelitis: a review. J. Clin. Sleep Med. 8, 719–728. 10.5664/jcsm.2276 23243408 PMC3501671

[B13] Johns M. W. (1991). A new method for measuring daytime sleepiness: the Epworth sleepiness scale. Sleep 14, 540–545. 10.1093/sleep/14.6.540 1798888

[B14] Karacan I. Thornby J. I. Anch M. Holzer C. E. Warheit G. J. Schwab J. J.. (1976). Prevalence of sleep disturbance in a primarily urban Florida County. Soc. Sci. Med. 10, 239–244. 10.1016/0037-7856(76)90006-8 968513

[B15] Kawamura A. Yoshiike T. Matsuo M. Kadotani H. Oike Y. Kawasaki M.. (2022). Comparison of the usability of an automatic sleep staging program via portable 1-channel electroencephalograph and manual sleep staging with traditional polysomnography. Sleep Biol. Rhythms. 10.1007/s41105-022-00421-5 PMC10899901 38468906

[B16] Kripke D. F. Simons R. N. Garfinkel L. Hammond E. C. (1979). Short and long sleep and sleeping pills. Is increased mortality associated? Arch Gen Psychiatry. 36, 103–116. 10.1001/archpsyc.1979.01780010109014 760693

[B17] Kryger M. H. Roth T. Dement W. C. (2022). Principles and Practice of Sleep Medicine, 6th edition. Amsterdam: Elsevier.

[B18] Landry G. J. Best J. R. Liu-Ambrose T. (2015). Measuring sleep quality in older adults: a comparison using subjective and objective methods. Front. Aging Neurosci. 7, 166. 10.3389/fnagi.2015.00166 26441633 PMC4561455

[B19] Liang Z. Chapa-Martell M. A. A. (2021). multi-level classification approach for sleep stage prediction with processed data derived from consumer wearable activity trackers. Front. Digit. Health. 3, 665946. 10.3389/fdgth.2021.665946 34713139 PMC8521802

[B20] Lichstein K. L. Means M. K. Noe S. L. Aguillard R. N. (1997). Fatigue and sleep disorders. Behav. Res. Ther. 35, 733–740. 10.1016/S0005-7967(97)00029-6 9256516

[B21] Lind M. J. Aggen S. H. Kirkpatrick R. M. Kendler K. S. Amstadter A. B. A. (2015). longitudinal twin study of insomnia symptoms in adults. Sleep 38, 1423–1430. 10.5665/sleep.4982 26132482 PMC4531410

[B22] Littner M. Hirshkowitz M. Kramer M. Kapen S. Anderson W. M. Bailey D.. (2003). Practice parameters for using polysomnography to evaluate insomnia: an update. Sleep 26, 754–760. 10.1093/sleep/26.6.754 14572131

[B23] Liu H. Shah S. Jiang W. (2004). On-line outlier detection and data cleaning. Comput. Chem. Eng. 28, 1635–1647. 10.1016/j.compchemeng.2004.01.009

[B24] Luca G. Haba Rubio J. Andries D. Tobback N. Vollenweider P. Waeber G.. (2015). Age and gender variations of sleep in subjects without sleep disorders. Ann. Med. 47, 482–491. 10.3109/07853890.2015.1074271 26224201

[B25] Maquet P. (2001). The role of sleep in learning and memory. Science 294, 1048–1052. 10.1126/science.1062856 11691982

[B26] Matsuo M. Masuda F. Sumi Y. Takahashi M. Yamada N. Ohira M. H.. (2016). Comparisons of portable sleep monitors of different modalities: potential as naturalistic sleep recorders. Front. Neurol. 7, 110. 10.3389/fneur.2016.00110 27471489 PMC4946159

[B27] Ministry of Health LaW (2019). The National Health and Nutrition Examination Survey. Available online at: https://www.mhlw.go.jp/stf/newpage_14156.html (accessed October 5, 2019).

[B28] Neu D. Mairesse O. Verbanck P. Linkowski P. Le Bon O. (2014). Non-REM sleep EEG power distribution in fatigue and sleepiness. J. Psychosom. Res. 76, 286–291. 10.1016/j.jpsychores.2014.02.002 24630178

[B29] Nofzinger E. A. Price J. C. Meltzer C. C. Buysse D. J. Villemagne V. L. Miewald J. M.. (2000). Towards a neurobiology of dysfunctional arousal in depression: the relationship between beta EEG power and regional cerebral glucose metabolism during NREM sleep. Psychiatry Res. 98, 71–91. 10.1016/S0925-4927(00)00045-7 10762734

[B30] Omichi C. Kadotani H. Sumi Y. Ubara A. Nishikawa K. Matsuda A.. (2022). The NinJaSleep Study Group. Prolonged sleep latency and reduced REM latency are associated with depressive symptoms in a Japanese Working Population. Int. J. Environ. Res. Public Health. 19, 2112. 10.3390/ijerph19042112 35206296 PMC8872621

[B31] Perlis M. L. Smith M. T. Andrews P. J. Orff H. Giles D. E. (2001). Beta/Gamma EEG activity in patients with primary and secondary insomnia and good sleeper controls. Sleep 24, 110–117. 10.1093/sleep/24.1.110 11204046

[B32] Pilcher J. J. Ginter D. R. Sadowsky B. (1997). Sleep quality versus sleep quantity: relationships between sleep and measures of health, well-being and sleepiness in college students. J. Psychosom. Res. 42, 583–596. 10.1016/S0022-3999(97)00004-4 9226606

[B33] Sharpe M. Wilks D. (2002). Fatigue. BMJ. 325, 480–483. 10.1136/bmj.325.7362.480 12202331 PMC1124000

[B34] Spiegel K. Leproult R. Van Cauter E. (1999). Impact of sleep debt on metabolic and endocrine function. Lancet 354, 1435–1439. 10.1016/S0140-6736(99)01376-8 10543671

[B35] Spiegelhalder K. Regen W. Feige B. Holz J. Piosczyk H. Baglioni C.. (2012). Increased EEG sigma and beta power during NREM sleep in primary insomnia. Biol. Psychol. 91, 329–333. 10.1016/j.biopsycho.2012.08.009 22960269

[B36] Stores G. Fry A. Crawford C. (1998). Sleep abnormalities demonstrated by home polysomnography in teenagers with chronic fatigue syndrome. J. Psychosom. Res. 45, 85–91. 10.1016/S0022-3999(98)00024-5 9720858

[B37] Susić V. Kovacévić-Ristanović R. (1980). Effects of restricted sleep with different exercise loads upon subsequent sleep. Arch. Int. Physiol. Biochim. 88, 1–13. 10.3109/13813458009080853 6155878

[B38] Svensson T. Chung U. I. Tokuno S. Nakamura M. Svensson A. K. (2019). A validation study of a consumer wearable sleep tracker compared to a portable EEG system in naturalistic conditions. J. Psychosom. Res. 126, 109822. 10.1016/j.jpsychores.2019.109822 31499232

[B39] Takami M. Kadotani H. Nishikawa K. Sumi Y. Nakabayashi T. Fujii Y.. (2018). Quality of life, depression, and productivity of city government employees in Japan: a comparison study using the Athens insomnia scale and insomnia severity index. Sleep. Sci. Pract. 2, 1–8. 10.1186/s41606-018-0024-0

[B40] Takegami M. Suzukamo Y. Wakita T. Noguchi H. Chin K. Kadotani H.. (2009). Development of a Japanese version of the Epworth Sleepiness Scale (JESS) based on item response theory. Sleep Med. 10, 556–565. 10.1016/j.sleep.2008.04.015 18824408

[B41] Tanaka M. Fukuda S. Mizuno K. Imai-Matsumura K. Jodoi T. Kawatani J.. (2008). Reliability and validity of the Japanese version of the Chalder Fatigue Scale among youth in Japan. Psychol. Rep. 103, 682–690. 10.2466/pr0.103.3.682-690 19320199

[B42] Torimoto K. Matsushita C. Itami Y. Iwamoto T. Owari T. Gotoh D.. (2022). Assessment of bladder function for stabilizing urinary volume overnight with recording of brain waves (ABSORB study). Low Urin Tract. Symptoms. 14, 72–77. 10.1111/luts.12412 34562069

[B43] Unruh M. L. Redline S. An M. W. Buysse D. J. Nieto F. J. Yeh J. L.. (2008). Subjective and objective sleep quality and aging in the sleep heart health study. J. Am. Geriatr. Soc. 56, 1218–1227. 10.1111/j.1532-5415.2008.01755.x 18482295

[B44] Van Cauter E. Plat L. (1996). Physiology of growth hormone secretion during sleep. J Pediatr. 128, S32–37. 10.1016/S0022-3476(96)70008-2 8627466

[B45] Waters F. Naik N. Rock D. (2013). Sleep, fatigue, and functional health in psychotic patients. Schizophr. Res. Treatment. 2013, 425826. 10.1155/2013/425826 23738067 PMC3659476

[B46] Yoshida M. Kashiwagi K. Kadotani H. Yamamoto K. Koike S. Matsuo M.. (2015). Validation of a portable single-channel EEG monitoring system. J. Oral Sleep Med. 1, 140–147.

