# Supplementary material for: Sleep-EEG-based parameters for discriminating fatigue and sleepiness
Source: Front Sleep. 2022 Oct 26;1:975415. doi: 10.3389/frsle.2022.975415 (PMC12713944; doi:10.3389/frsle.2022.975415)
Supplement: Supplementary file 1 [file Data_Sheet_1.docx]

Table S1: Chalder Fatigue Scale (original and Japanese versions)

| 1 | Do you have problems with tiredness? |
| --- | --- |
|  | 疲労（つかれた感じ）のためにこまることは？ |
| 2 | Do you need to rest more? |
|  | もっと休みたいと思うことは？ |
| 3 | Do you feel sleepy or drowsy? |
|  | 眠くなったり、ボーっとすることは？ |
| 4 | Do you have problems starting things? |
|  | 何かを始めるのに問題があることは？ |
| 5 | Do you start things without difficulty but get weak as you go on? |
|  | やり始めは問題ないのに続けるうちに気力がなくなっていくことは？ |
| 6 | Are you lacking in energy? |
|  | 活力がないと思うことは？ |
| 7 | Do you have less strength in your muscles? |
|  | 筋力低下を感じることは？ |
| 8 | Do you feel weak? |
|  | 自分のからだが弱々しいと感じることは？ |
| 9 | Do you have difficulty concentrating? |
|  | なかなか集中できないことは？ |
| 10 | Do you have problems thinking clearly? |
|  | 考えがうまくまとまらないことは？ |
| 11 | Do you make slips of the tongue when speaking? |
|  | 話そうとして、舌が回らなくなることは？ |
| 12 | Do you find it more difficult to find the correct word? |
|  | 話そうとして、適当な言葉が見つからないことは？ |
| 13 | How is your memory? |
|  | 記憶力はどうですか？ |
| 14 | Have you lost interest in the things you used to do? |
|  | 今までやってきたことに興味を失ったことは？ |

| Parameter | Description |
| --- | --- |
| TIB | Time in bed; duration from start to end of a recording. |
| SL | Sleep latency: duration from bedtime to sleep onset. |
| SLS3 | Stage N3 sleep latency; interval from sleep onset to first appearance of N3 sleep. |
| SLSR | REM sleep latency: interval between sleep onset and first appearance of REM sleep. |
| SPT | Sleep period time; duration between sleep onset and final awakening. |
| TST | Total sleep time; amount of actual sleep time. |
| TS1 | Time in N1. |
| TS2 | Time in N2. |
| TS3 | Time in N3. |
| TSR | Time in REM. |
| %SW | Percentage of Wake based on SPT. |
| %S1 | Percentage of N1 based on SPT. |
| %S2 | Percentage of N2 based on SPT. |
| %S3 | Percentage of N3 based on SPT. |
| %SR | Percentage of REM based on SPT. |
| ARnum | Number of mid arousals occurring in SPT. |
| ARI | Arousal index: frequency [number/hour] of mid arousals based on SPT |
| WASO | Wake time after sleep onset; total time spent awake during SPT. |
| ARnum2h | ARnum two hours before final awakening. |
| ARI2h | ARI two hours before final awakening. |
| WASO2h | WASO two hours before final awakening. |
| TSC1 | Duration of first sleep cycle (SC1). |
| TSC2 | Duration of second sleep cycle (SC2). |
| SCnum | Number of total sleep cycles. |
| SCavg | Average duration of all sleep cycles. |
| SC1ratio | Ratio of TSC1 to SCavg. |
| SC2ratio | Ratio of TSC2 to SCavg. |
| SE | Sleep efficiency; ratio of TST to TIB. |

Table S2: Sleep Parameters derived from time-domain analysis

| Parameter | Description |
| --- | --- |
| maxVal | Maximum value of power index. |
| maxT | Timing with the maximum value of power index from the period start. |
| maxTratio | Ratio of maxT to the length of the period. |
| minVal | Minimum value of power index. |
| minT | Timing with the minimum value of power index from the period start. |
| minTratio | Ratio of minT to the length of the period. |
| Avg | Average of power index. |
| Std | Standard deviation of power index. |
| Total | Sum of power index. |

Table S3: Sleep parameters derived from frequency-domain analysis
